# Total Phenolic Content and Antioxidant Activity of Different Types of Chocolate, Milk, Semisweet, Dark, and Soy, in Cerebral Cortex, Hippocampus, and Cerebellum of Wistar Rats

**DOI:** 10.1155/2015/294659

**Published:** 2015-11-16

**Authors:** Niara da Silva Medeiros, Roberta Koslowsky Marder, Mariane Farias Wohlenberg, Cláudia Funchal, Caroline Dani

**Affiliations:** Department of Biochemistry, Research Center, Centro Universitário Metodista IPA, 90420-060 Porto Alegre, RS, Brazil

## Abstract

Chocolate is a product consumed worldwide and it stands out for presenting an important amount of phenolic compounds. In this study, the total phenolic content and antioxidant activity in the cerebral cortex, hippocampus, and cerebellum of male Wistar rats when consuming different types of chocolate, including milk, semisweet, dark, and soy, was evaluated. The total polyphenols concentration and antioxidant activity in vitro by the method of DPPH radical-scavenging test were evaluated in chocolate samples. Lipid peroxidation (TBARS), protein oxidation (carbonyl), sulfhydryl groups, and activity of SOD enzyme in cerebral cortex, hippocampus, and cerebellum of rats treated or not with hydrogen peroxide and/or chocolate were also evaluated. The dark chocolate demonstrated higher phenolic content and antioxidant activity, followed by semisweet, soy, and milk chocolates. The addition of chocolate in the diet of the rats reduced lipid peroxidation and protein oxidation caused by hydrogen peroxide. In the sulfhydryl assay, we observed that the levels of nonenzymatic defenses only increased with the chocolate treatments The SOD enzyme activity was modulated in the tissues treated with the chocolates. We observed in the samples of chocolate a significant polyphenol content and an important antioxidant activity; however, additional studies with different chocolates and other tissues are necessary to further such findings.

## 1. Introduction

Chocolate is characterized as solid dispersion of cocoa mass, sugar, additives, cocoa butter, lecithin, and flavonoids; there may be variations between types; for example, in milk chocolate, fat, protein milk, and lactose are added to the formulation [1]. Cocoa should be present in the formulation of all types of chocolate in a proportion of at least 25%. Cocoa is known for being rich in at least three types of flavonoids: procyanidins, catechins, and epicatechins, even if the chocolate manufacturing and handling may cause loss up to 80% of flavonoids originating from cocoa beans [2, 3]. The amount of cocoa liquor used in the manufacture of chocolate is directly linked to the final amount of polyphenols present in it. Another characteristic that is attributed to the amount of liquor in the formulation is its bitter taste; for this reason the one that contains higher content of cocoa liquor is called bitter chocolate [4, 5].

Considering consumers' increased concern in getting a healthier lifestyle, foods with antioxidant properties have been widely studied, since they demonstrate an important role in combating oxidative stress [6]. Oxidative stress occurs in the body when there is an imbalance between oxidant molecules, including the Reactive Oxygen Species (ROS) and antioxidants, thus generating cell damage [7]. Among the compounds with antioxidant properties most consumed in the diet, we highlight the polyphenols, including flavonoids that are present in many fruits such as grapes and citrus fruits and in vegetables such as spinach and broccoli and also abundantly found in cocoa beans and, consequently, chocolate [8, 9].

Clinical studies have suggested that flavonoids in cocoa may act beneficially in preventing cardiovascular diseases through its effects in reducing blood pressure, improving endothelial function, increasing nitric oxide, reducing platelet aggregation and blood lipids, and decreasing insulin resistance [9, 10]. Antimutagenic and antiproliferative effects of tumor cells were also connected to the antioxidant activity of flavonoids in chocolate [11–13]. Furthermore, chocolate is often consumed under emotional stress because it induces positive effects on mood [14].

Currently in the Brazilian markets, there are several types of chocolate, divided according to the quantity of cocoa liquor, dark chocolate being the one with the greatest quantity [1]. Soy chocolate has, instead of milk, soy in its composition and its flavor resembles semisweet chocolate. In this kind, the amount of cocoa liquor can vary [1]. However, prior to this study there were no studies comparing the in vitro antioxidant activity of different types of chocolates present on the market: milk, dark, semisweet, and soy chocolates. In this context, the present study aims to establish the comparison among some of the leading brands of chocolates traditionally sold in the Brazilian market and to evaluate the protective action of these chocolates on some parameters of oxidative stress in cerebral cortex, hippocampus, and cerebellum of Wistar rats.

## 2. Materials and Methods

### 2.1. Obtaining Samples

For this study, four different samples of chocolates available in the market were acquired, denominated MC (milk chocolate), DC (dark chocolate), SMC (semisweet chocolate), and SC (soy chocolate). The dark and semisweet chocolates contained on their packaging information on the percentage of cocoa in their composition, which was 67% and 43%, respectively.

### 2.2. Sample Preparation

The samples were prepared according to the technique described by Vinson et al. [15] and modified by Pimentel et al. [16]. 100 mg chocolate aliquots were defatted through multiple extractions with hexane. The samples were incubated at 35°C overnight and then 50 mg of them were mixed with a solution composed of 50 mL of hydrochloric acid (HCl) 1.2 M with the addition of 50% aqueous methanol. The final solution is then heated in a water bath at 90°C for 2 hours with stirring at every 30 minutes and the extract obtained is filtrated.

### 2.3. Determination of Total Polyphenols

The extracts obtained by the technique of Vinson et al. [15] were used for the analysis of total polyphenols using 1000 *μ*L of Folin-Ciocalteu reagent, diluted with distilled water (1 : 9), which was added to 100 mL of chocolate extract or distilled water that was used for control. The samples were left at room temperature for 20 minutes for color development. As standard, a solution of catechin (0.2 mg/mL of methanol) was utilized and read by UV-visible spectrophotometer, 750 nm.

### 2.4. Scavenging Activity of 1,1-Diphenyl-2-picrylhydrazyl (DPPH) Radicals

The antioxidant activity of chocolates was evaluated by DPPH radical-scavenging test, where 200 *μ*L samples were mixed with 800 *μ*L of Tris-HCl 100 mM solution pH 7.0. 1000 *μ*L of ethanolic solution of DPPH 250 mM was added to this mixture and the tubes were maintained for 20 minutes protected from light; absorbance then measured, at UV-visible spectrophotometer, 517 nm. Trolox was used as positive control. Water was used as negative control. The percentage of DPPH inhibition was calculated using the following formula: Scavenging capacity (%) = 100 − [(absorbance of sample − absorbance of sample blank) × 100/(absorbance of control) − (absorbance of control blank)]. The IC_50_ values were calculated from the graph plotted as inhibition percentage against the concentration. IC 50%, meaning 50% of the DPPH radical, was the sample amount required to be scanned.

### 2.5. Tissue Preparation

10 Wistar rats, 10 days old, were used in this study. The animals were obtained from the animal facility of Centro Universitário Metodista IPA (South Brazil). Assays were performed as described by Leipnitz et al. [17]. Animals were euthanized by decapitation without anesthesia and the brain was quickly extracted. Cerebral cortex, hippocampus, and cerebellum were dissected and immediately homogenized in potassium chloride (KCl) 1.5% to maintain the integrity of the cells. This study was approved by the Ethics Commission for Animal Use (CEUA) of the Centro Universitário Metodista IPA under protocol number 004/2011.

### 2.6. In Vitro Assay: Treatment and Evaluation of Antioxidant Activity

For further analysis, 720 *μ*L of homogenate was treated with the extract obtained from the chocolates (90 *μ*L). After incubation for 30 minutes at 37°C, H_2_O_2_ (90 *μ*L) was added to the mix and then the samples were again incubated for 30 minutes in a water bath at 37°C. After incubation levels of lipid peroxidation (TBARS), protein oxidation (carbonyl), total sulfhydryl groups (SH groups), and activity of superoxide dismutase (SOD) were evaluated. Lipid peroxidation was monitored by the formation of thiobarbituric acid reactive species (TBARS) during an acid-heating reaction [18]. The oxidative damage of proteins was measured by determination of carbonyl groups based on the reaction of dinitrophenylhydrazine [19].

The antioxidant activity was determined by the level of total sulfhydryl groups and the antioxidant activity enzyme SOD. The total sulfhydryl groups were determined in an assay that is based on the reduction of 5,5-dithiobis (2-nitrobenzoic acid) (DTNB) which generates a yellow derivative, whose absorbance is measured in a spectrophotometer at 412 nm [20]. Antioxidant capacity of the SOD was determined using a spectrophotometer by measuring the inhibition of adenocromo autocatalytic at 480 nm (spectrophotometer model UV-1700, Shimadzu, Kyoto, Japan) [21].

### 2.7. Statistical Analysis

Data were subjected to analysis of variance (ANOVA) followed by Tukey's post-test, with significance level of 0.05. The relationship between variables was determined using Pearson's correlation. All analysis were performed using the statistical program Statistical Package for the Social Sciences (SPSS) version 17.0 (International Business Machines Corporation, New York, NY, USA).

## 3. Results

Identifying new sources of phenolic compounds in the diet is of great interest to public health due to its antioxidant activity potential. It is known that chocolate is a major source of these compounds; however, not all types of chocolate are equal sources of polyphenols. In our study, the different types of chocolates tested showed statistically significant differences (*p* < 0.05): dark chocolate (60% cocoa) the richest in this parameter, followed by semisweet chocolate (43% cocoa) (Table 1).

The antioxidant activity of chocolates is demonstrated in DPPH IC_50_ (Table 1), with the dark chocolate showing the highest antioxidant activity, followed by soy and semisweet chocolate. The dark chocolate showed the same antioxidant activity as the positive control (Trolox). We observed a negative correlation between the DPPH IC_50_ and polyphenol contents (*R* = −0914, *p* < 0.01), analyzed in chocolates.

**Figure 1:**
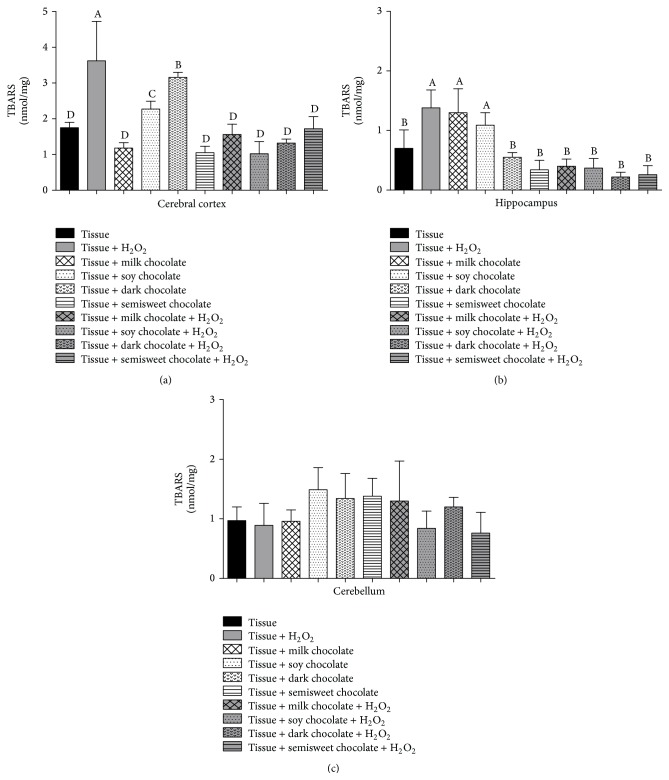
Levels of lipid peroxidation (TBARS) in cerebral cortex (a), hippocampus (b), and cerebellum (c) of Wistar rats treated with different chocolate types with or without hydrogen peroxide. Different letters show statistical difference between them (*p* < 0.05).

Lipid peroxidation levels (TBARS) increased in cerebral cortex in soy and dark chocolate, but this increase was lower than H_2_O_2_, *p* < 0.05 (Figure 1(a)). Hippocampus tissue also presented an increase in TBARS levels with the addition of H_2_O_2_ and this increase was prevented in all treatments with chocolate (*p* < 0.05). However, in the hippocampus when TBARS were evaluated, both chocolates, milk and soy, were able to increase these levels equaling to the value found on the tissue when H_2_O_2_ was added (Figure 1(b)). Lastly, in the cerebellum, no statistical difference was observed in this parameter (Figure 1(c)).

**Figure 2:**
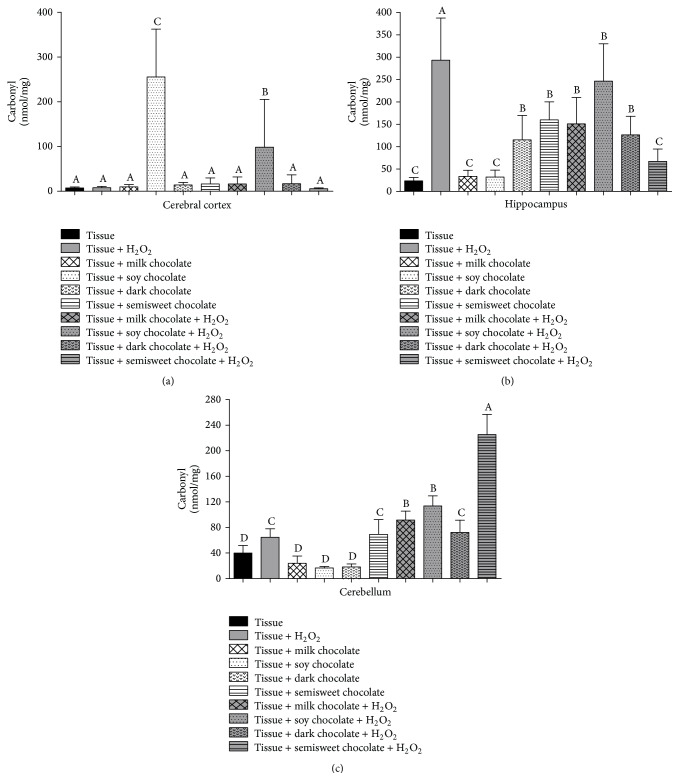
Levels of protein oxidation (carbonyl) in cerebral cortex (a), hippocampus (b), and cerebellum (c) of Wistar rats treated with different chocolate types with or without hydrogen peroxide. Different letters show statistical difference between them (*p* < 0.05).

For levels of protein oxidation, we observed that H_2_O_2_ in the cerebral cortex was not capable of inducing the oxidative damage (Figure 2(a)). Nevertheless, in hippocampus, H_2_O_2_ induced oxidative damage in protein, but milk, soy, and dark chocolates prevented the damage (Figure 2(b)). In cerebellum, H_2_O_2_ was able to cause increased levels of carbonyls when compared to the control. The milk, soy, and dark chocolates alone did not increase this parameter level. However, chocolate with H_2_O_2_ was not able to prevent oxidative damage to protein in this tissue (Figure 2(c)).

**Figure 3:**
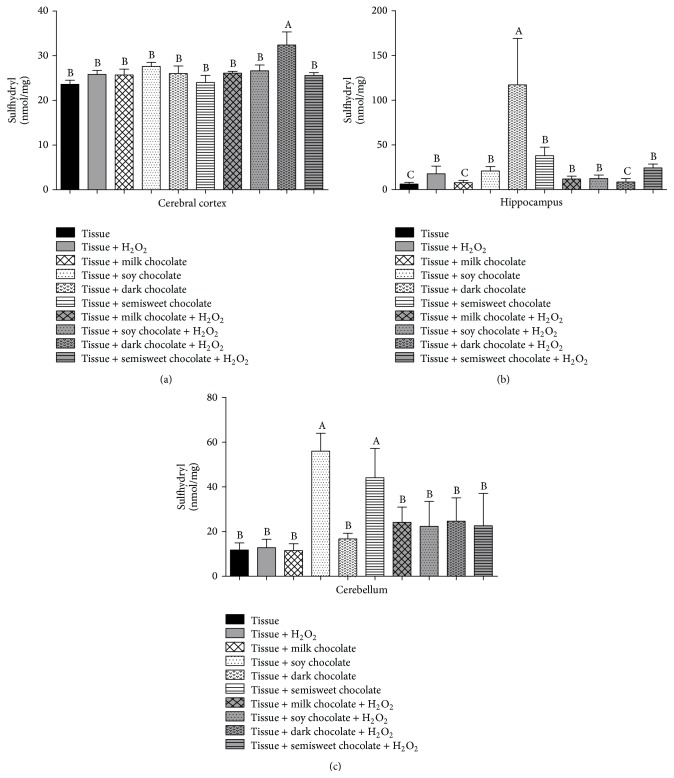
Antioxidant activity by sulfhydryl groups in cerebral cortex (a), hippocampus (b), and cerebellum (c) of Wistar rats treated with different chocolate types with or without hydrogen peroxide. Different letters show statistical difference between them (*p* < 0.05).

In the sulfhydryl assay (SH groups), in the cerebral cortex, we observed increased levels only in the treatment with dark chocolate added to H_2_O_2_ (*p* < 0.05), no significant change being seen in the other treatments (Figure 3(a)). In the hippocampus, the content of sulfhydryl grouping increased in the tissue where only dark chocolate was added. The opposite was observed when H_2_O_2_ was added to the tissues along with the chocolates, which showed a decrease of these levels (Figure 3(b)). However, in the cerebellum, groups treated only with soy and semisweet chocolates were able to increase the levels of sulfhydryl (Figure 3(c)).

**Figure 4:**
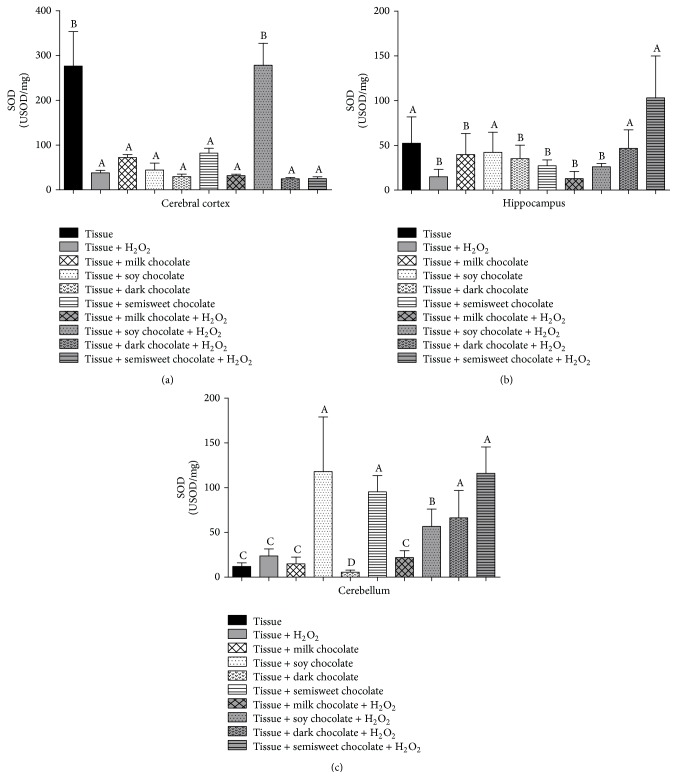
Antioxidant activity of the SOD enzyme in cerebral cortex (a), hippocampus (b), and cerebellum (c) of Wistar rats treated with different chocolate types with or without hydrogen peroxide. Different letters show statistical difference between them (*p* < 0.05).

The activity of SOD enzyme is shown in Figure 4. We observed that addition of H_2_O_2_ to the tissue reduced SOD activity of the cerebral cortex and this reduction was maintained with the addition of all the chocolates except soy chocolate where we evidence a rise in this enzyme, equivalent to the control. This reduction of SOD was also observed in all the treatments with tissue where the different chocolates were added (Figure 4(a)). In the hippocampus, the SOD activity increased with soy chocolate placed along the tissue; this increase was also observed in the presence of dark and semisweet chocolate in tissue exposed to H_2_O_2_ (Figure 4(b)). When observing the cerebellum, the activity of SOD increased in groups treated only with soy and semisweet chocolate; the same evidence was observed in the groups treated with semisweet and dark chocolate and H_2_O_2_ (Figure 4(c)).

## 4. Discussion

Chocolate is a product that stands out for presenting an important amount of phenolic compounds, with values higher than the polyphenols found in red wine, for example [8]. As the main influential factor in the content of polyphenols in chocolate, we can quote its content of cocoa liquor [16] present in the samples analyzed in our study. Dark chocolate had 67% of cocoa liquor in its composition and the semisweet chocolate had 43% higher amounts of liquor than milk and soy chocolates. These data corroborate the study conducted by Miller et al. [5] with different samples of chocolates commercialized in the United States, where the polyphenol content was also higher in dark chocolate. The geographical origin, plant variety, climate, soil type, and planting region are factors that can influence the content of polyphenols in cocoa [22]. The various processing steps that transform cocoa in chocolate can also change the phenolic content of the final product [22].

Phenolic compounds are gaining visibility due to their recognized antioxidant capacity, delaying the rate of oxidation through mechanisms such as the inhibition of ROS and metal complexation [23]. The antioxidant activity of a certain compound is measured by the reactivity of electron donor hydrogen and the ability to displace or stabilize an unpaired electron [24]. In our study, the antioxidant activity of chocolate is expressed in DPPH IC_50_, where the dark chocolate has the highest antioxidant activity, followed by semisweet, soy, and milk chocolate. Corroborating the findings of the high antioxidant activity of dark chocolate, we have the study of Hermann et al. [25] where the consumption of dark chocolate caused an increased plasma antioxidant capacity concomitant with improved vasodilation in young smokers, confirming the role of chocolate flavonoids in reduction of ROS.

In addition, our study demonstrated that soy chocolate showed less amount of total polyphenols when compared to semisweet chocolate; however, both obtained the same antioxidant activity. Soy products have important content of isoflavones (genistein and daidzein); these have high antioxidant power requiring less quantity to have a good antioxidant potential [26].

In addition, studies in the literature attribute the antioxidant activity of different products to their phenolic composition [27, 28]. This fact was also observed in our study, where a negative correlation between the amount of total polyphenols and the DPPH IC_50_ was observed, since the higher the phenolic content, the lower the DPPH IC_50_, in other words, the higher the antioxidant activity. As dark chocolate presented the highest concentration of total polyphenols, it showed the lowest DPPH IC_50_ (Table 1), meaning that a smaller sample amount was required to scan 50% of DPPH^•^ radical than the amount of the other types of chocolates analyzed. The low antioxidant activity of milk chocolate, among the chocolate types tested, can be explained since this chocolate is the one that has greater amount of sugar in its preparation. The negative influence of sugar on antioxidant activity was demonstrated in a study by Franke et al. [29] with orange juice where juice with high sugar showed a reduction in the capacity to scavenge superoxide anions as well as the decrease in lipid peroxidation.

In almost all analyses of our study, the addition of H_2_O_2_ to the tissue induced oxidative damage, as demonstrated in TBARS and carbonyl. This happens because although the H_2_O_2_ is not a free radical, it is a metabolite of oxygen extremely deleterious with a long life and capable of crossing lipid layers and it can react with the membrane and proteins, mainly with Fe^++^, provoking a significant damage [30]. As the damage was reversed by the various treatments with chocolate, the protection demonstrated in our study corroborates the findings of Fraga et al. [31] that proved that there was a decrease in markers of oxidative stress in young football players who consumed 105 g of milk chocolate for 14 days.

In this present study, a modulating effect of chocolates on the activity of SOD enzyme was demonstrated. It was found that the activity decreased with the addition of H_2_O_2_, and this fact is due to increased oxidative stress caused by such compound; decreases in SOD are due to irreversible inactivation by its product H_2_O_2_ [32]. However, the chocolates were unable to reverse this reduction of H_2_O_2_ in the cerebral cortex. Regarding the increase in SOD enzyme activity in the treatment with soy chocolate and H_2_O_2_ in the same tissue, it may be due to the significant presence of isoflavones in this type of chocolate. The increased activity of this enzyme was possibly induced by the presence of these compounds, as it was verified in a study conducted by Leng et al. [33], where the isoflavones increased antioxidant activity in the liver of rats with nonalcoholic steatohepatitis. The SOD activity also decreased with the addition of H_2_O_2_ in the hippocampus and cerebellum, where we observe that mainly the dark and semisweet chocolate were able to prevent the reduction caused by the damage inductor.

## 5. Conclusion

The data obtained in this study show a significant concentration of total polyphenols and an important antioxidant activity in the chocolates analyzed. These results are relevant, since chocolate is a food commonly consumed in everyday diet of the world population because of its favored palatability. However, more studies with different chocolates, as well as assessing different tissues, are needed to corroborate our findings.

## Figures and Tables

**Table 1:** Total polyphenols and DPPH radical-scavenging activity of different types of chocolate.

Samples	Total polyphenols	DPPH IC_50_
	(*μ*mol catechin/g)	(*μ*g/mL)
Milk chocolate	28.44 ± 1.68^a^	49.18 ± 2.47^a^
Soy chocolate	35.18 ± 0.53^a^	23.69 ± 1.50^b^
Semisweet chocolate	45.67 ± 2.95^b^	32.60 ± 2.07^b^
Dark chocolate	106.81 ± 3.28^c^	10.43 ± 1.39^c^
Trolox		9.71 ± 2.13^c^

IC_50_ = concentration that inhibits 50% of the initial concentration of DPPH radical.

Different letters show statistical difference between them (*p* < 0.05).
